# Protein S Deficiency Unmasked by Young-Onset Stroke: A Case Report

**DOI:** 10.7759/cureus.78846

**Published:** 2025-02-11

**Authors:** Nithish Nanda Palanisamy, Bala Vignesh Kalyanasundaram, Nandha Kumar Selvam

**Affiliations:** 1 General Internal Medicine, Coimbatore Medical College, Coimbatore, IND; 2 Internal Medicine, Coimbatore Medical College, Coimbatore, IND; 3 Emergency Medicine, Kovai Medical Center and Hospital, Coimbatore, IND

**Keywords:** acute stroke management, cerebrovascular accident (stroke), emergency management pathways, hereditary protein s deficiency, young-onset stroke

## Abstract

This report presents a rare case of ischemic stroke in a 16-year-old female, which was later diagnosed as being caused by protein S deficiency, a hereditary thrombophilia. Stroke at a young age is uncommon, and identifying the underlying cause is critical for proper management. The patient presented with right-sided weakness and slurred speech, with MRI confirming an ischemic infarct. Initial treatment with aspirin and atorvastatin was provided, but further investigations revealed low protein S levels, leading to the diagnosis. She was then managed with low-molecular-weight heparin (LMWH), followed by oral warfarin, resulting in significant neurological recovery. While inherited thrombophilias are uncommon causes of arterial strokes, this case underscores the importance of early identification and targeted treatment of such rare etiologies. Prompt initiation of anticoagulation therapy with LMWH followed by oral warfarin led to significant neurological improvement. This report highlights the need for thorough thrombophilia workup, individualized management, and the role of anticoagulation in improving outcomes, even in resource-limited settings.

## Introduction

Young stroke is a cerebrovascular disease pertaining to the age group of less than 45 years, whose incidence has been increasing [1]. Young survivors of cerebrovascular disease have increased incidence of depression, dependence, unemployment, and being single, which affect their quality of life and result in significant morbidity [2-4]. Here, we report the case of a 16-year-old adolescent female presenting with acute-onset weakness. Early diagnosis and treatment of the root cause have improved prognosis, for which this patient could be cited as an example. India reported a high incidence of young stroke (15-40 years of age) of about 25%, as reported by Abraham et al. [5]. According to Munts et al., idiopathic coagulation disorders were found in about 25% of young stroke patients [6]. They also reported that a confirmed coagulation disorder was associated with large-vessel disease [6]. Thus, ruling out acquired and inherited causes of thrombophilia plays an important role in the diagnosis and prognosis of young stroke patients. Protein S deficiency is one of the inherited/acquired thrombophilia which results in preferential venous thromboembolism [7]. The action of activated protein C on activated factor V and activated factor VIII is facilitated by protein S, thus functioning as an anticoagulant [7]. A study about the prevalence of thrombophilia by Carod-Artal et al. in Brazilian stroke patients revealed protein S deficiency (11.5% versus 5.5%) and protein C deficiency (0.76% versus 1%) in about 130 young and 200 elderly patients, respectively [8]. This case report focuses on the challenges in the diagnosis and treatment of inherited thrombophilias with a special focus on inherited protein S deficiency.

## Case presentation

A 16-year-old female studying in grade 10 came to the emergency department (ED) with complaints of an inability to use the right upper and lower limbs for the past six hours. She developed sudden difficulty in lifting her right hand, writing using her right hand, and walking. Maximal weakness was present at the time of presentation with no progression. She had a history of slurring of speech. There was no history of sensory loss, altered sensorium, fever, seizures, headaches, vomiting, blurring of vision, involuntary movements, and loss of consciousness. The patient was a firstborn child of a non-consanguineous marriage. The pregnancy was uneventful with term vaginal delivery. There was no neonatal intensive care unit admission. She attained menarche two years back and had a regular menstrual cycle of 7/30 days with normal flow. Family history was insignificant, and no other comorbidities were present.

Upon arrival, she was conscious and oriented and her vital parameters were SpO_2_ of 98% in room air, respiratory rate of 18 breaths/minute, heart rate of 92 beats/minute, blood pressure of 110/70 mmHg, random blood sugar of 104 mg/dL, and temperature of 97.6°F.

**Figure 1:**
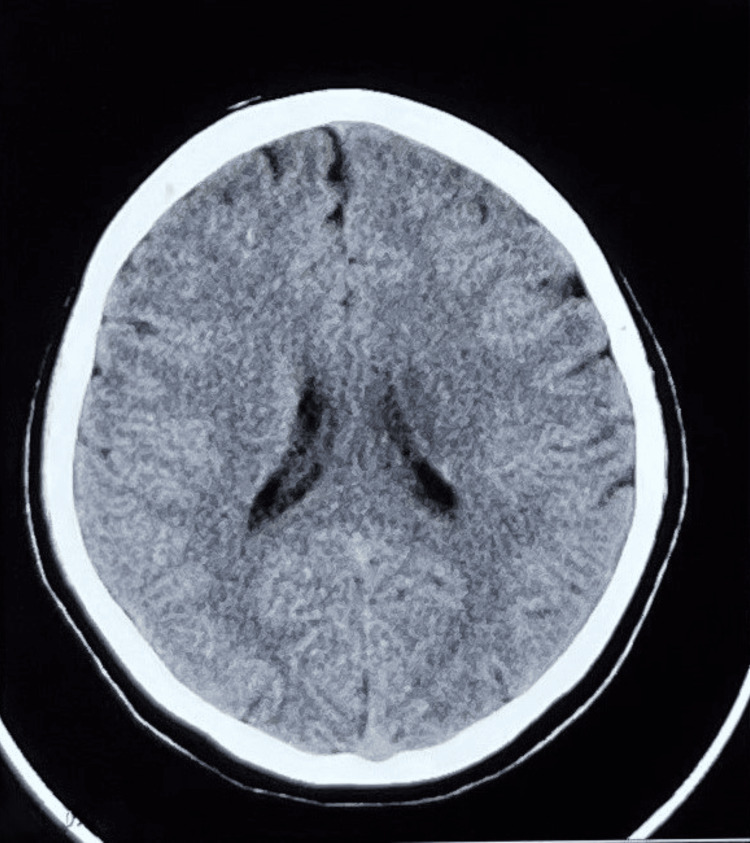
Non-contrast CT of the brain. Non-contrast CT of the brain is the first step to rule out hemorrhage in patients presenting with acute-onset focal neurological deficits. The CT revealed no evidence of hemorrhage and, hence, the focal neurological deficit was due to an ischemic phenomenon.

Neurological examination revealed slurred speech with an otherwise normal higher mental function, slight deviation of the angle of the mouth to the right side, and loss of nasolabial fold with an inability to hold air in the mouth, suggestive of upper motor neuron facial nerve palsy. Other cranial nerve examinations were normal. The tone was normal bilaterally, with power being 3/5 in the right and 5/5 in the left upper and lower limbs. The patient had a positive Babinski sign on the right side. The cerebellar examination was normal. Other systems were also normal. A non-contrast CT scan of the brain revealed no evidence of hemorrhage (Figure 1).

**Figure 2:**
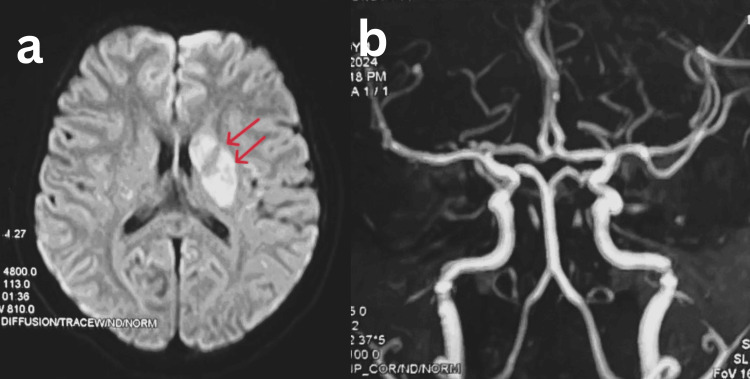
Magnetic resonance imaging with magnetic resonance angiography of the brain. (a) MRI revealed diffusion restriction in the left corona and lentiform region. (b) MRA showed no evidence of large-vessel occlusion.

Her routine blood investigation, including complete blood count, renal function test, liver function test, serum proteins, and coagulation, was normal. She was non-reactive for human immunodeficiency virus and hepatitis B. An electrocardiogram showed normal findings. As the presentation to the ED was late (more than 4.5 hours), tissue plasminogen activator therapy was not started. MRI of the brain with magnetic resonance angiography (MRA) was ordered to localize the infarct and rule out large-vessel occlusion. MRI revealed diffusion restriction in the left corona and lentiform region (Figure 2). MRA showed no evidence of large-vessel occlusion, following which she was put on aspirin and atorvastatin (Figure 2).

As the patient presentation was at a young age without any risk factors for atherosclerosis, workup for thrombophilia was ordered, along with cerebrovascular Doppler and transthoracic echocardiography. Cerebrovascular Doppler was normal, while ultrasonography of the abdomen and pelvis revealed bilateral polycystic ovaries. Echocardiography was normal with no clots and an ejection fraction of 64%.

Additional thrombophilia workup of functional assays for antithrombin III, protein S deficiency, polymerase chain reaction for factor V Leiden mutation, and testing for antiphospholipid antibodies and homocysteine levels revealed reduced protein S function and protein C at borderline (Table 1). A diagnosis of ischemic stroke due to protein S deficiency was made and the patient was managed with intravenous low-molecular-weight heparin (LMWH), followed by oral warfarin along with aspirin, atorvastatin, and folic acid. The patient was discharged on the seventh day of the hospital stay, with the right-sided power having improved to 4/5. The patient and her family were advised of the need for regular physiotherapy, family support, and follow-up.

**Table 1:** Thrombophilia workup findings. Thrombophilia workup of functional assays for antithrombin III, protein S deficiency, polymerase chain reaction for factor V Leiden mutation, and testing for antiphospholipid antibodies and homocysteine levels revealed reduced protein S function and borderline protein C.

Parameter	Result	Reference range
Protein C activity	69.28	65–140 IU/dL (enzyme-linked fluorescent assay)
Protein S activity	34.0	60–140 IU/dL (photo-optical clot detection)
Homocysteine	15.1	No folate supplementation children <15 years: <10
Folic acid	9.2	2.5–20 ng/mL
Vitamin B12	400	200–800 pg/mL

## Discussion

Inherited deficiency is caused by *PROS1* gene mutation, which is autosomal dominant in inheritance [7-9]. Common acquired causes of this condition include vitamin K deficiency, liver diseases, oral anticoagulant therapy, acute illness, pregnancy, use of oral contraceptives, estrogen therapy, and nephrotic syndrome [7]. Our patient was more likely to have had congenital protein S deficiency, as there were no findings suggestive of any other cause. However, the absence of thrombotic diseases in first-degree relatives raises clinical suspicion. This dilemma is addressed preferentially with clinical correlation owing to the inability to undergo multiple laboratory tests due to financial constraints.

There is preferential venous thrombosis over arterial thrombosis in protein S-deficient patients [10-12]. In a study by Wiesel et al., only 14 patients had arterial thrombotic accidents involving the central nervous system or myocardium out of 105 patients with protein S deficiency [10]. There is no supportive evidence in other studies to attribute arterial infarcts to protein S deficiency [10-13]. In the absence of a substantial correlation, warfarin prophylaxis can be questioned in patients with acquired thrombophilias. Even though the correlation between protein S deficiency and stroke requires further investigation, the study by Wang et al. reported protein S mutation to be the primary cause of thrombosis in a family [14]. A case report by Sheetal et al. also demonstrated the need for anticoagulant prophylaxis in women with protein S deficiency presenting as recurrent ischemic stroke [15]. Our patient presented with arterial thrombosis of the cerebrovascular system as the initial presentation of protein S deficiency. She was initiated on LMWH and then changed to oral warfarin. This led to significant improvement in her neurological deficits, with power increasing to 4/5 in the affected limb, and the patient was able to walk with support. Hence, the presence of protein S deficiency cannot be completely ignored due to the absence of correlation, with anticoagulation playing an important role. Direct oral anticoagulants (apixaban and rivaroxaban) have been preferred in patients with ischemic stroke with protein S deficiency [15,16]. Due to the financial constraints of the patient, oral warfarin therapy was continued.

## Conclusions

Protein S deficiency, whether inherited or acquired, remains a significant risk factor for thrombotic events, predominantly venous thrombosis. However, its association with arterial thrombosis, particularly ischemic stroke, is less substantiated and requires further investigation. In our patient, the clinical presentation of cerebrovascular arterial thrombosis highlights the potential role of protein S deficiency as an underlying etiology despite the absence of significant family history or supportive evidence from cohort studies. Prompt initiation of anticoagulation with LMWH, followed by oral warfarin, led to marked neurological improvement. While direct oral anticoagulants are emerging as preferred agents in such cases, economic constraints necessitated the continued use of warfarin in this patient. This case underscores the importance of individualized management in protein S deficiency, with careful clinical correlation guiding treatment decisions to optimize patient outcomes.
